# GSH and Ferroptosis: Side-by-Side Partners in the Fight against Tumors

**DOI:** 10.3390/antiox13060697

**Published:** 2024-06-06

**Authors:** Yulang Jiang, Christian Glandorff, Mingyu Sun

**Affiliations:** 1 Shuguang Hospital, Shanghai University of Traditional Chinese Medicine, Shanghai 201203, China; albert_jylss@163.com (Y.J.); christian@glandorff.de (C.G.); 2 Internal Medicine in Chinese Medicine, Shanghai University of Traditional Chinese Medicine, Shanghai 201203, China; 3 Key Laboratory of Liver and Kidney Diseases, Institute of Liver Diseases, Shuguang Hospital, Shanghai University of Traditional Chinese Medicine, Shanghai 201203, China; 4 University Clinic of Hamburg at the HanseMerkur Center of TCM, 20251 Hamburg, Germany

**Keywords:** GSH, ferroptosis, lipid peroxidation, oxidative stress, tumors

## Abstract

Glutathione (GSH), a prominent antioxidant in organisms, exhibits diverse biological functions and is crucial in safeguarding cells against oxidative harm and upholding a stable redox milieu. The metabolism of GSH is implicated in numerous diseases, particularly in the progression of malignant tumors. Consequently, therapeutic strategies targeting the regulation of GSH synthesis and metabolism to modulate GSH levels represent a promising avenue for future research. This study aimed to elucidate the intricate relationship between GSH metabolism and ferroptosis, highlighting how modulation of GSH metabolism can impact cellular susceptibility to ferroptosis and consequently influence the development of tumors and other diseases. The paper provides a comprehensive overview of the physiological functions of GSH, including its structural characteristics, physicochemical properties, sources, and metabolic pathways, as well as investigate the molecular mechanisms underlying GSH regulation of ferroptosis and potential therapeutic interventions. Unraveling the biological role of GSH holds promise for individuals afflicted with tumors.

## 1. Introduction

**Figure 1:**
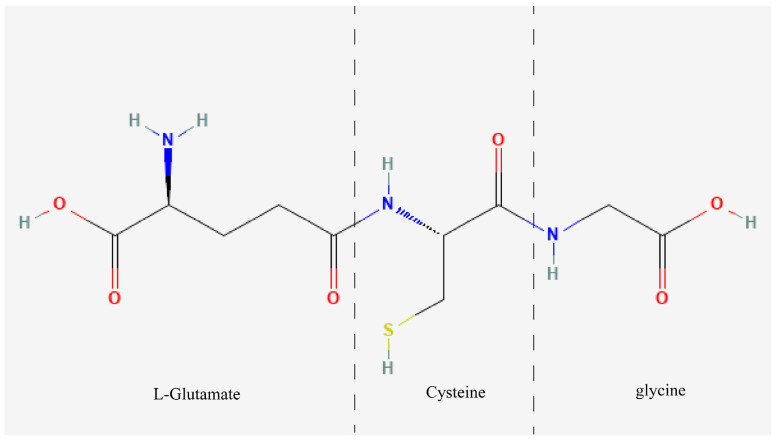
Chemical structural formula of GSH. One molecule of GSH is obtained from one molecule of L-glutamic acid, one molecule of cysteine, and one molecule of glycine by a dehydration condensation reaction.

GSH, a tripeptide composed of glutamate, cysteine, and glycine, is synthesized through sequential catalysis by glutamate-cysteine ligase (GCL) and glutathione synthetase (GSS) enzymes [1,2] (Figure 1). It is the most abundant non-protein thiol in eukaryotic cells, primarily existing in reduced and oxidized forms as GSH and glutathione disulfide (GSSG). Within the cytoplasm, GSH is predominantly found in its reduced state, with oxidized GSH representing a small fraction, estimated to be in the range of 10,000:1–50,000:1 [3]. These two forms of GSH can interconvert during intracellular redox reactions [4].

The metabolic pathways of GSH play a crucial role in maintaining its content homeostasis, which in turn serves as a fundamental molecule for regulating cellular redox homeostasis [5]. The regulation of amino acids that synthesize GSH raw materials, the content and activity of GSH synthase, the subcellular spatiotemporal distribution of GSH, and the mechanism of GSH catabolism and degradation can significantly affect GSH content and influence human GSH homeostasis [6]. The GSH/SLC7A11/GPx4 axis is the most important defense axis of ferroptosis, and SLC7A11, as the upstream of the ferroptosis pathway, serves as the raw material for the synthesis of GSH through the uptake of cystine, whereas GSH, through the action of GPx4, is able to reduce lipid peroxides on the plasma membrane to lipids and alcohols, thus protecting cells from ferroptosis [7,8]. More than that, GSH is also involved in the regulation of cell metabolism, proliferation, and gene expression. GSH is also involved in the post-translational modification of certain intracellular proteins, which regulates signal transduction and redox balance [9].

This paper provides a concise overview of the comprehensive pathways and regulatory mechanisms involved in GSH metabolism. Additionally, considering the significant impact of GSH on ferroptosis, this paper also discusses the pharmacological agents and small molecule compounds that modulate GSH levels to regulate ferroptosis and potentially treat tumors.

## 2. GSH Metabolism

The level of GSH within the organism is maintained in a state of dynamic equilibrium through the intricate regulation of processes such as synthesis, utilization, distribution, uptake, and decomposition [10].

### 2.1. GSH Macrocycle in the Body

**Figure 2:**
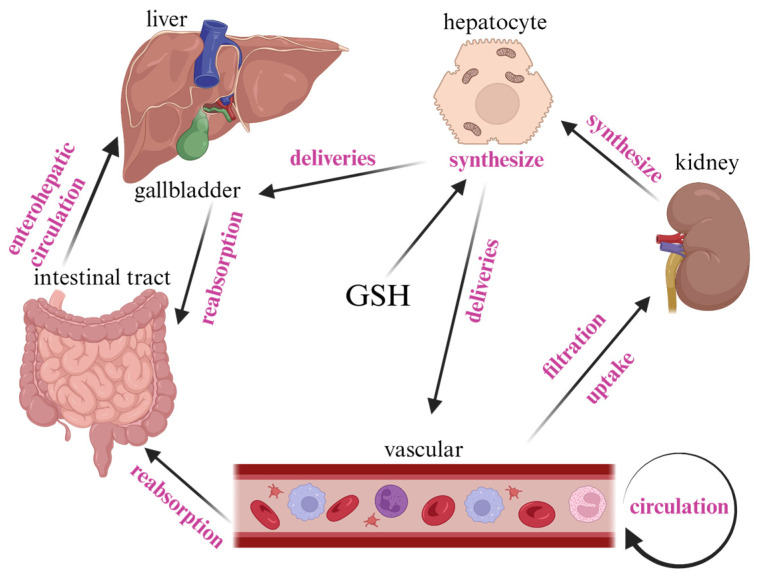
Schematic diagram of the general circulation of GSH in the body. GSH is synthesized by hepatocytes and partly transported to the gallbladder and partly to the blood to participate in the circulation; GSH entering the gallbladder is reabsorbed by the small intestine, and GSH in the blood is metabolized by filtration in the kidneys.

GSH is produced primarily by hepatocytes in the liver. A fraction of the GSH synthesized in the liver is eliminated into the bile via the bile ducts, while the remainder is transported into the bloodstream through specific proteins on hepatocytes. GSH discharged into the bile undergoes hydrolysis to cysteine by gamma-glutamyltransferase (γ-GGT) in bile duct and intestinal epithelial cells, subsequently being reabsorbed and utilized by the small intestine for GSH synthesis. This newly formed GSH is then reabsorbed and utilized by the liver, thus completing the enterohepatic cycle of GSH. GSH is absorbed by the kidneys through two primary mechanisms upon entering the bloodstream. The first mechanism involves filtration through the glomerulus, with approximately half of the GSH filtered and subsequently catabolized by GGT and dipeptidase (DP) enzymes located at the brush border of renal tubular cells. The cysteine produced from this catabolism is then reabsorbed by the renal proximal tubule for the ongoing synthesis of GSH. The second mechanism involves the intact uptake of GSH in its tripeptide form at the basement membrane of renal proximal tubular cells through specific uptake transporters. The renal uptake of GSH plays a crucial role in its utilization by the kidneys [11]. A portion of GSH is also excreted out of the body in the urine [12,13,14,15,16] (Figure 2).

### 2.2. GSH Source

#### 2.2.1. Exogenous Intake

The supplementation of exogenous GSH presents itself as a straightforward and efficient method for modulating intracellular GSH levels [17]. While some studies indicate that the in vivo levels of GSH and antioxidant capacity remain unaffected by both short-term and long-term oral GSH administration [18], other research findings suggest that oral GSH intake can lead to a dose-dependent increase in in vivo GSH levels and a reduction in associated oxidative stress markers [19,20]. Therefore, the effectiveness of exogenous glutathione supplementation requires further evaluation through larger-scale clinical cohort studies [21,22].

#### 2.2.2. Endogenous Synthesis

Regarding its chemical composition, GSH is a tripeptide composed of L-glutamic acid, cysteine, and glycine. The biosynthesis of GSH involves the utilization of these three amino acids as substrates and is carried out through two ATP-dependent enzymatic reactions. Glutamate can be acquired by the body through uptake via the glutamate transporter on the cell membrane, or through transmembrane Na^+^-dependent alanine-serine-cysteine transporter type-2 (ASCT2) uptake of glutamine [23], followed by hydrolysis by glutaminase 1 (GLS1) and GSL2 to generate glutamate [24,25]. Cysteine, a dimeric amino acid composed of two cysteine molecules connected by a disulfide bond, is a constituent of various proteins and glutathione within living organisms [26]. While cysteine is directly absorbed by the ASC, its extracellular presence is vulnerable to oxidation in the oxidative extracellular environment, leading to its rapid conversion to cystine [27,28]. As a result, the primary source of intracellular cysteine is derived from the transsulfuration of methionine. In the transsulfuration metabolic pathway, homocysteine undergoes condensation with serine to generate cystathiones under the catalytic action of cystathionine β-synthase (CBS). Subsequent hydrolysis of cystathiones by cystathionine gamma-lyase (CTH) yields cysteine [29,30]. Intracellular cysteine can be converted from extracellular cystine through the glutamate-cystine reverse transporter, also known as the Xc-system, leading to rapid reduction of cystine to cysteine within the cytosol. Intracellular glycine is acquired through direct uptake via the glycine transporter on the cell membrane [31]. 

The synthesis of glutathione, facilitated by the availability of all amino acid substrates within the cell, involves a two-step enzymatic reaction catalyzed by GCL and glutathione synthetase (GSS) [5,32]. The GCL enzyme complex is comprised of a catalytic subunit (GCLC) and a regulatory subunit (GCLM), with GCLC responsible for inducing significant conformational changes and GCLM serving as a molecular chaperone to maintain the stability of the protein complex [33,34]. This complex facilitates the synthesis of γ-glutamyl-cysteine by linking cysteine and glutamate. Specifically, GCL catalyzes the dehydration condensation of cysteine with glutamate in the initial step, resulting in the production of γ-glutamyl-cysteine. It is noteworthy that this reaction generates a γ-carboxylic group, in contrast to the typical formation of an α-carboxylic group. In the subsequent stage, facilitated by the enzyme GSS, γ-glutamylcysteine experiences dehydration condensation with glycine to produce glutathione [35,36]. The presence of GSS results in a minimal concentration of γ-glutamylcysteine, thereby establishing that the expression of GCL, enzyme activity, and cysteine levels serve as the determining factor in the rate of GSH synthesis under normal physiological circumstances. Additionally, these factors are subject to negative feedback regulation by the intracellular GSH levels [37,38].

Therefore, the pharmacological modulation of specific amino acid transporters that regulate intracellular cysteine levels and modulate the activity of the catalytic enzyme GCS shows promise in the regulation of GSH levels in living organisms. Further comprehensive research into the genetic mechanisms that govern GSH content and activity is warranted.

### 2.3. Distribution of GSH

The involvement of the glutathione-centered redox system in redox signaling networks plays a crucial role in regulating cell growth, development, and oxidative defense [10]. Both exogenously ingested and intracellularly synthesized GSH must be transported efficiently to specific tissues at appropriate times to exert its beneficial effects. Consequently, investigating the temporal and spatial distribution of GSH is essential for optimizing its functionality [39].

Initially, GSH is predominantly synthesized by hepatocytes and localized within the liver. Nevertheless, for optimal utilization of GSH’s regulatory capabilities, its distribution extends to various cell types, albeit with varying concentrations. Furthermore, apart from intercellular discrepancies in GSH levels, temporal, and spatial variations in GSH distribution within homogenous cellular environments further underscore the intricate and precise regulation of GSH within the human body.

In a broad sense, glutathione is predominantly localized within the cytoplasm, but it is also present in diverse subcellular organelles such as the nucleus, mitochondria, and endoplasmic reticulum. The distribution of glutathione within these organelles exhibits considerable variation. The dynamic distribution of glutathione at varying concentrations over time intervals plays a crucial role in establishing a redox environment, modulating cellular metabolism, and signaling pathways. The regulation of redox balance in various cellular compartments, including the nucleus, mitochondria, endoplasmic reticulum, and extracellular environment, is intricately linked to the presence and function of glutathione.

#### 2.3.1. Cytoplasm

Glutathione is synthesized solely in the cytoplasm of mammalian cells, with approximately 85% of the synthesized glutathione being retained in this cellular compartment [40]. Within the cytoplasm, glutathione predominantly exists in its reduced form, with its oxidized form, GSSG, representing a minor fraction. Experimental evidence has shown that cytoplasmic GSH can interact with Fe^2+^ to form an Fe-S complex, which subsequently binds to members of the poly(rC)-binding protein (PCBP) family to generate the PCBPI- Fe-GSH complex [41]. The cytoplasm has been reported to have a high concentration of GSH, up to 10 mmol/L, while the concentration of GSSG in the cytoplasm is on a nanomolar scale. The predominance of GSH in the cytosol does not conflict with its distribution in other subcellular organelles. Due to the absence of glutathione synthase in these organelles, GSH must be transported from the cytoplasm. In addition to the aforementioned reduced and oxidized forms of glutathione, other forms may also be present. GSH is known to interact with proteins, forming protein-GSH mixed disulfides (PSSG) that are typically found in the cytosol on a temporary basis, except for periods of oxidative stress.

#### 2.3.2. Mitochondria

The mitochondrial pool of reduced GSH comprises approximately 10–15% of the overall intracellular GSH pool and is predominantly in a reduced state [42]. The concentration of GSH within each mitochondrion is comparable to that in the cytosol, and there is no discernible concentration gradient within the inner mitochondrial membrane space. Due to the absence of glutathione synthetase, mitochondria are unable to produce GSH intrinsically. However, they are capable of acquiring GSH from the cytoplasm, which is facilitated by the permeability of the two layers of the inner mitochondrial membrane and the outer mitochondrial membrane. The presence of pore proteins on the outer mitochondrial membrane enables molecules smaller than 5 kDa to traverse freely, allowing GSH to diffuse directly through the outer mitochondrial membrane into the intermembrane space, where its concentration equilibrates with that of the cytoplasm [43]. Nevertheless, the translocation of GSH across the inner mitochondrial membrane into the mitochondrial matrix necessitates the involvement of two anion transporters, namely the dicarboxylate carrier (DCC) and the 2-oxoglutarate carrier (OGC). These transporters facilitate the exchange of specific anions within the mitochondrial matrix with GSH in the interstitial space of the membrane, thereby enabling the transport of GSH into the matrix [44]. It is widely recognized that mitochondria serve as the primary location for aerobic respiration and the generation of reactive oxygen species (ROS). Superoxide dismutase and catalase within mitochondria play a role in reducing ROS; however, the presence of a sufficient quantity of GSH is necessary to maintain redox balance and mitigate oxidative damage caused by free radicals, particularly due to the limited abundance of catalase. Glutathione peroxidase (GPx) facilitates the oxidation of GSH to GSSG and the reduction of H_2_O_2_ generated by superoxide dismutase to H_2_O, thereby minimizing free radical-induced cellular damage. Additionally, GSH reductase aids in the regeneration of GSH for subsequent cycles of antioxidant defense [45].

#### 2.3.3. Nucleus

Despite the low concentration of GSH in the nucleus, research has demonstrated the significant impact of GSH on the cell cycle, with indications that cells primed for division exhibit elevated levels of nuclear GSH. Consequently, investigating the correlation between GSH and the cell cycle could enhance our comprehension of cellular physiology and metabolic pathways. Furthermore, it is widely accepted that low and moderate levels of ROS can stimulate mitosis and support cell growth, while excessive ROS can result in DNA damage and mutations, ultimately leading to oxidative stress. Additionally, GSH is essential for maintaining DNA repair and expression within the nucleus. During RNA reduction, GSH serves as a key hydrogen donor, facilitating the conversion of RNA to DNA and promoting DNA synthesis.

#### 2.3.4. Endoplasmic Reticulum

The endoplasmic reticulum, a complex network within the cytoplasm, is responsible for a range of essential functions such as protein biosynthesis, folding, translocation, glycosylation, and disulfide bond formation [46,47]. Within the endoplasmic reticulum, the GSH:GSSG ratio can reach levels as high as 1–15:1 [48]. In times of oxidative stress, GSSG can engage in disulfide bond exchange reactions with sulfhydryl groups of proteins, resulting in the formation of protein mixed disulfide bonds, known as PSSG. These PSSG molecules can then further interact with sulfhydryl groups of other proteins to create protein disulfide bonds. The rate of these reactions is significantly reduced in the absence of catalysis by enzymes, such as protein disulfide isomerase (PDI), which is a crucial enzyme abundant in the endoplasmic reticulum where protein folding takes place [49]. Consequently, the high GSSG/GSH ratio observed in the vesicles of the endoplasmic reticulum can be attributed to the presence of PDI. The formation of disulfide bonds plays a crucial role in protein synthesis within the endoplasmic reticulum, serving to maintain the highly oxidized environment necessary for the organelle to carry out its functions effectively. Alterations in the redox state of the endoplasmic reticulum have a significant impact on the process of disulfide bond formation, which involves the oxidation of GSH to GSSG [50].

#### 2.3.5. Other Organelles

**Figure 3:**
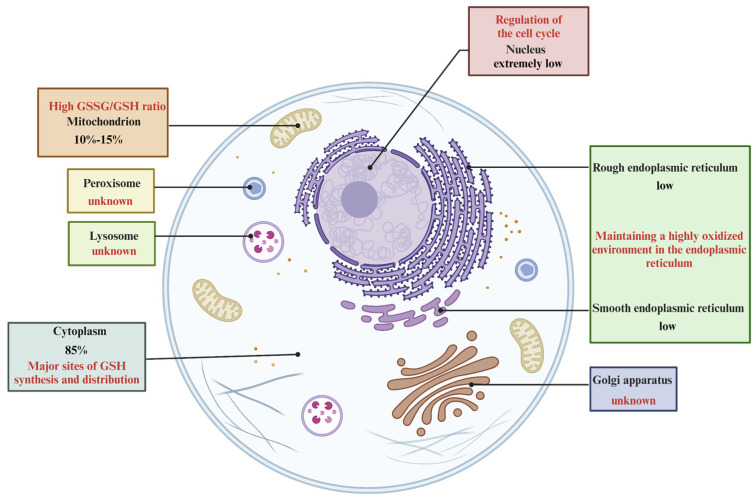
Distribution, function, and characterization of GSH in various organelles. The ingredient amino acids for the synthesis of GSH are transported into the cell via transporters on the surface of the cell membrane or are obtained intracellularly by transformation. After intracellular acquisition of GSH substrate amino acids, γ-glutamine cysteine is generated by GCL, and further synthesized by GS enzymatic reaction. GSH can be catabolized into substrate amino acids by intracellular GGT enzymes or degraded intracellularly by Chac1/2.

Further research is needed to investigate the distribution of GSH in various intracellular organelles, including Golgi, lysosomes, and ribosomes, as well as the specific biological roles they may play [51,52] (Figure 3).

#### 2.3.6. Tumor Tissue Heterogeneity of GSH/GSSG

Heterogeneity in cancer tissues is a well-recognized phenomenon, and it extends to the metabolic profile of tumor cells. Tumor cells within the same tumor mass often exhibit varying metabolic strategies to support their proliferation, survival, and adaptation to the tumor microenvironment [53]. This metabolic heterogeneity can be observed in the levels and ratios of various metabolites, including GSH/GSSG.

Recent studies have shown that cancer tissues exhibit heterogeneity in GSH/GSSG levels and ratios. This heterogeneity can be observed both spatially within the tumor mass and temporally during tumor progression. Some cancer cells may have higher levels of GSH to cope with oxidative stress and promote proliferation, while others may have lower levels due to increased oxidative stress or metabolic reprogramming.

One study used liquid chromatography and mass spectrometry to detect a significantly lower GSH/GSSG ratio in breast cancer cells compared to normal breast epithelial cells [54]. In a piglet model of high-grade spinal cord glioma (SCG), the GSH/GSSG ratio was reduced at the tumor margins compared to the tumor center, and oxidative stress was increased [55]. Heterogeneity in GSH/GSSG ratios also exists between different cancer types; in general, GSH levels are lower and GSSG levels are higher in breast cancer [56]. In a 2010 study, researchers found a significantly lower GSH/GSSG ratio in liver cancer tissues, which may be related to the oxidative stress state of liver cancer cells [57]. Lower GSH levels in tumor tissues than in normal tissues and higher levels of GSSG resulting in a lower GSH/GSSG ratio were observed in patients with non-small cell lung cancer, and this change may be associated with the proliferation and invasiveness of lung cancer cells [58]. However, it is important to note that GSH/GSSG changes in tumors are complex and varied, depending on tumor type, stage, treatment and individual patient differences, etc.

This metabolic heterogeneity in cancer tissues has important implications for cancer treatment. The heterogeneity of metabolites and GSH/GSSG levels in cancer tissues suggests that a single therapeutic agent may not be effective against all tumor cells. Therefore, combination therapies targeting multiple metabolic pathways may be more effective in treating cancer.

In summary, GSH is found throughout the cell, primarily in the cytoplasm, where it functions to protect against oxidative stress. Additionally, GSH within individual organelles contributes to maintaining redox homeostasis within the cell.

### 2.4. GSH Degradation

Glutathione is subject to catabolic processes via two principal pathways: exogenous metabolism, which occurs extracellularly, and endogenous metabolism, a pathway recently discovered to take place within the cytoplasm.

#### 2.4.1. Exogenous Metabolic Pathway

The distinctive structure of GSH arises from the condensation of glutamate and cysteine, resulting in the formation of a tertiary carboxyl group (γ carboxyl group) rather than the typical α carboxyl group [59]. Despite the resistance of most enzymes to hydrolyze the tertiary carboxyl group, GGT stands out as the sole enzyme capable of catalyzing this specific reaction. GGT is a cell surface enzyme typically localized at the apices of ducts and glands. In patients with tumors, GGT exhibits elevated expression levels that are broadly distributed across the cell membrane [60]. The enzymatic activity of GGT enables cells to regulate intracellular levels of reduced GSH, thereby enhancing resistance to the toxic effects of promoting compounds and facilitating response to proliferative signals induced by oncogenic stimuli. GGT catalyzes the hydrolysis of the gamma–glutamyl bond in extracellular oxidized and reduced forms of GSH [61]. GSH functions as a reservoir of cysteine and acts as a semiessential amino acid for the biosynthesis of GSH in conditions of limited cysteine availability. Following translocation to sites activated by GGT, GSH may undergo degradation to yield L-glutamate and cysteinylglycine or cystine and glycine. These breakdown products can subsequently be released as glutamate, cysteine, cystine, and glycine through the catalytic action of DP on the cellular membrane. The amino acids or dipeptides generated through cytolytic catabolism can be taken up by the cell for the synthesis of GSH [62]. The GGT facilitates the uptake of cysteine by the tumor, leading to elevated levels of intracellular GSH. This enables the tumor to regulate its redox equilibrium in response to the production of reactive oxygen species induced by pro-oxidant treatments, thereby evading cell death triggered by oxidative stress [63].

#### 2.4.2. Endogenous Metabolic Pathways

The degradation of glutathione has traditionally been understood to occur exclusively within the non-cytoplasmic pool, as the enzyme responsible for this process, GGT, is localized to either the plasma membrane in mammals and bacteria or the vesicular membrane in yeast and plants, where it acts on the extracellular or vesicular pool. However, recent research has identified several new enzymes involved in glutathione degradation within the cytoplasmic lysosome, shedding new light on this biochemical pathway. Various enzymes, such as DUG enzymes in yeast and fungi, ChaC1 enzymes in higher eukaryotes, ChaC2 enzymes spanning from bacteria to humans, and RipAY enzymes in select bacteria, have been identified with diverse roles ranging from fundamental cellular maintenance to stress response mechanisms [64,65]. These enzymes are implicated in crucial biological processes including embryonic neurodevelopment and pathogenesis. This study specifically examined the GHS-degrading enzymes that are pertinent to human physiology, with intracellular degradation of GSH facilitated by ChaC1 and ChaC2 proteins acting as cation transport regulators [66]. In contrast to GGT, members of the ChaC family enzymatically degrade reduced GSH localized in the cytoplasm, without affecting oxidized GSH [67]. This enzymatic process involves the hydrolysis of GSH to yield cysteinyl-glycine and 5-oxoproline. Subsequent enzymatic reactions further break down cysteinyl-glycine to cysteine and glycine, and 5-oxoproline to L-glutamate. These resulting amino acids can then participate in the re-synthesis of GSH [68].

**Figure 4:**
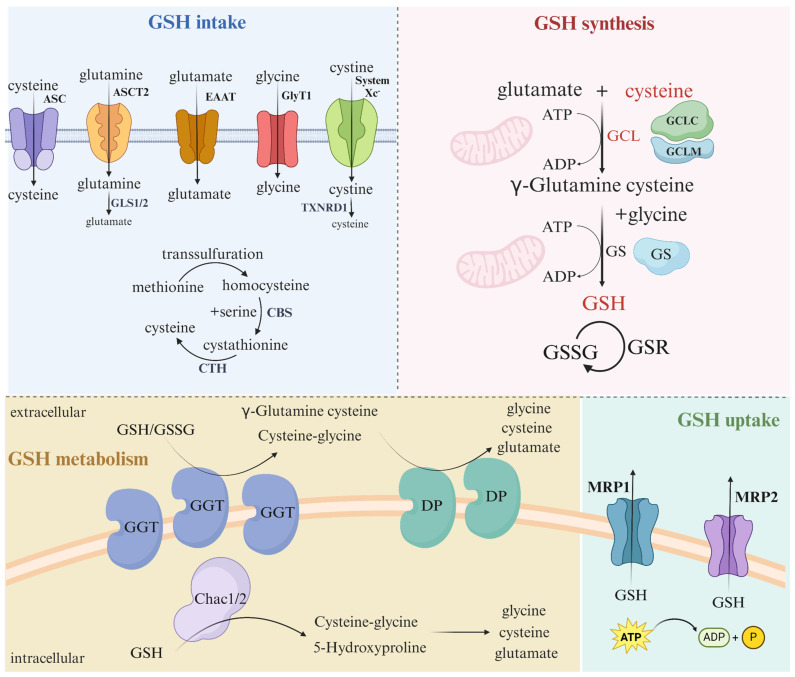
Synthesis and metabolism of GSH. Almost all GSH is synthesized in the cytoplasm, with about 85% distributed in the cytoplasm and mainly in the reduced form of GSH. The amount of GSH in the cytoplasm then determines the GSH content of the entire cell. About 10–15% of GSH is distributed in the mitochondria, which is the main site of ROS production, and therefore a certain amount of GSH is needed to avoid oxidative damage to the cell by free radicals. The content of GSH in the nucleus is extremely low, but it does play an irreplaceable role in the regulation of cellular mitosis. The endoplasmic reticulum also contains GSH, and the ratio of GSH:GSSG can be as high as 1–15:1, which is the material basis for the formation of endoplasmic reticulum in a highly oxidized environment, and the highly oxidized environment is a necessary condition for the endoplasmic reticulum to perform its functions.

Several studies have indicated that a decrease in the rate of GSH metabolism can result in the buildup of excessive GSH within the body, potentially facilitating tumor growth and invasion in specific physiological or pathological circumstances [69]. The complexity of GSH metabolism allows for the regulation of GSH levels to prevent the unchecked accumulation of GSH and subsequent cellular damage [2]. Maintaining a stable range of GSH levels is crucial for neutralizing harmful substances like hydrogen peroxide and lipid peroxides, thereby safeguarding cells against oxidative stress. Changes in GSH homeostasis significantly impact cellular physiology and have been implicated in various pathological conditions such as diabetes, neurodegenerative diseases, and cancer. The importance of glutathione degradation in cellular function exceeds previous understanding, as differential regulation and specificity of enzymes acting on distinct pools of glutathione can lead to diverse cellular outcomes [12] (Figure 4).

### 2.5. GSH Uptake

GSH content can be affected not only by the synthetic system, but also by the membrane transporter protein-MRP1 in cells. Multidrug resistance protein 1 (MRP1) is an ATP-binding cassette (ABC) protein, the overexpression of which produces cellular resistance to a wide range of anticancer drugs and regulates redox homeostasis [70]. MRP1 is responsible for the translocation of intracellular GSH or other complexes bound to GSH out of the cell, one of the important mechanisms for maintaining stable intracellular GSH levels. GSH can bind to many substances to form conjugates that can subsequently be recognized and transported by MRP1 [71]. Specifically, the expression level and activity status of MRP1 has an important effect on intracellular GSH content. When MRP1 expression is upregulated or its activity is enhanced, it can more effectively translocate GSH or its conjugates out of the cell, thereby reducing intracellular GSH levels, which may make cancer cells more sensitive to oxidative therapy [72]. In contrast, inhibition of MRP1 expression elevates intracellular GSH levels, thereby providing antioxidant protection to tumor cells during cancer development, which may also account for the insensitivity of some tumor cells to ferroptosis inducers [73]. A recent study also noted that in ischemia-reperfusion injury [74], GSH is released during IR in an MRP1-dependent manner and induces ferroptosis during disease progression, and inhibition of GSH release can effectively attenuate myocardial injury caused by IR.

In the coming years, these downstream effects may be revealed in greater detail and will lead to a better understanding and appreciation of glutathione degradation.

## 3. Molecular Mechanisms Involved in the Regulation of Ferroptosis by GSH

Early research has demonstrated the significance of cystine in facilitating normal cell growth and proliferation. In the absence of cystine in the culture medium, cellular growth is hindered [75]. Subsequent studies have revealed that deprivation of cystine in the culture medium results in a swift reduction of intracellular glutathione levels, ultimately leading to cellular demise. It is currently understood that ferroptosis, a form of cell death, is reliant on GSH levels and is initiated by the accumulation of lipid ROS [76]. This process is characterized by the buildup of lipid peroxides, highlighting the significance of impaired lipid peroxide scavenging resulting from inadequate or inhibited activity of endogenous antioxidant mechanisms in the occurrence of ferroptosis [77].

ROS are endogenous cellular metabolites that play essential roles in various physiological and biochemical processes [78]. Maintaining a delicate balance between ROS production and elimination is crucial for the preservation of a conducive physiological environment [79]. Oxidative stress occurs when the equilibrium between oxidative and antioxidative mechanisms is disturbed [80,81]. Cells typically have the ability to manage moderate levels of oxidative stress; however, excessive oxidative stress surpassing the cell’s antioxidant capabilities can result in harm to lipids, proteins, and DNA, ultimately leading to cell death [82]. Two primary methods of inducing oxidative stress in cells include elevating ROS levels to accelerate oxidative damage kinetics and impairing the antioxidant defense system to compromise the cell’s protective mechanisms [83]. 

The glutathione system serves as a vital antioxidant defense mechanism against ROS, playing a critical role in the maintenance of cellular redox equilibrium and normal physiological functions by regulating the balance of redox-regulated molecules such as NAD/NADH, NADP/NADPH, and GSH/GSSG ratios [3,84,85].

The GPx family of proteins play a crucial role as endogenous antioxidant enzymes in the body [86,87], with GPx4-mediated enzymatic antioxidant reactions serving as a key mechanism for mitigating lipid peroxidation and acting as a primary endogenous inhibitor of ferroptosis [88,89]. Numerous studies have demonstrated that the inhibition of GPx4 results in elevated levels of ROS and lipid peroxidation [90], while increased expression of GPx4 leads to a reduction in ROS levels and the inhibition of cellular ferroptosis. GPx4 is capable of utilizing reduced GSH as a cofactor to facilitate the reduction of lipid peroxidation, converting lipid hydroperoxides (LOOH) to lipid alcohols (LOH) and thereby impeding the process of ferroptosis [91]. The oxidation of GSSG produced in this process is then reduced to reduced GSH by GSH reductase and NADPH/H [92]. The catalytic site of this reaction is located on the selenocysteine residue of GPx4, such that the binding of RAS-selective lethal small molecule (RSL3) to the ligand of the nucleophilic portion of selenocysteine in the active site of GPx4 directly deactivates or hinders GPx4 activity [93]. Furthermore, selenium is essential for the preservation of GPx4 activity. In instances of selenium deficiency, GPx4 becomes deactivated due to the irreversible peroxidation of thiocyanate at its catalytic active site, leading to heightened cellular susceptibility to oxidative harm [94,95,96].

The regulation of intracellular GSH levels is primarily governed by the transport of cystine through the Xc-system [76]. This system, comprised of the sodium-independent cystine/glutamate inverse transporter 7 (SLC7A11), which consists of a light-chain subunit (SLC7A11) and a heavy-chain subunit (SLC3A2), efficiently facilitates cystine transport by the coordinated action of both subunits. While most amino acids can be transported by multiple transporters, the Xc-system is unique in its specificity for cystine transport. Among these proteins, SLC7A11 predominantly functions as a transporter with high specificity for cystine and glutamate, while SLC3A2 primarily acts as a chaperone protein crucial for maintaining the stability of SLC7A11 [6]. Consequently, the inhibition or knockdown of SLC7A11 results in decreased intracellular cysteine levels, finally impacting GSH levels and promoting ferroptosis [97].

**Figure 5:**
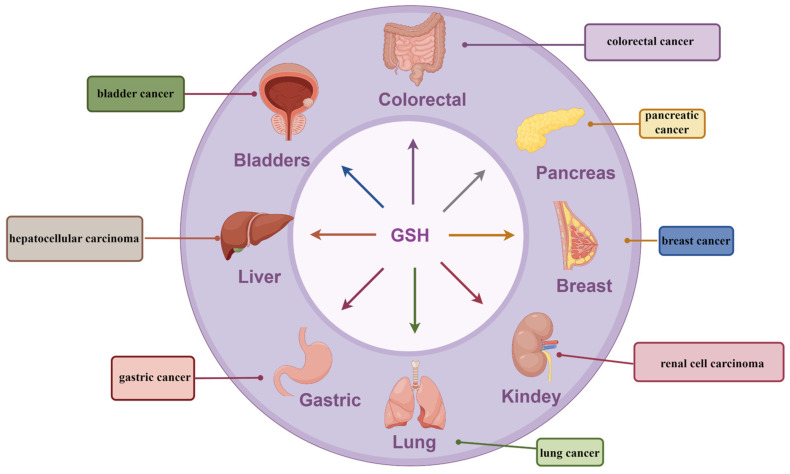
Tumors associated with ferroptosis in which GSH is involved. GSH has been associated with tumors in a variety of human systems, involving ferroptosis in several organs, including the liver, kidney, gastrointestinal tract, pancreas, lungs, bladder, and mammary glands.

In summary, GPx4 serves as a critical modulator of ferroptosis, while the GSH antioxidant system plays a pivotal role in the regulation of ferroptosis under the control of GPx4, with the Xc-system acting as the central regulator of GSH levels (Figure 5).

## 4. GSH Metabolism-Based Ferroptosis Regulating Molecules, Drugs in Tumors

Prior research has demonstrated a correlation between heightened levels of GSH and resistance to chemotherapy in tumors, with the glutathione antioxidant defense system potentially rendering cancer cells resistant to current chemotherapeutic treatments. Consequently, a deficiency in GSH within cancer cells may enhance their susceptibility to oxidative stress and chemotherapy, indicating that a modest reduction in GSH levels could serve as an efficacious approach to heighten the responsiveness of cancer cells to chemotherapy [4].

**Figure 6:**
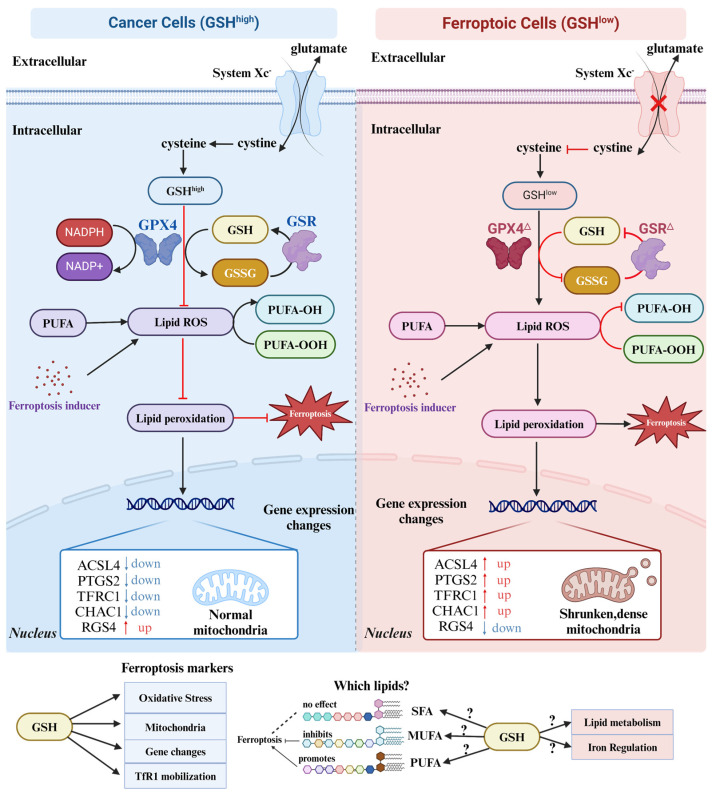
Molecular mechanisms involved in the regulation of ferroptosis by GSH. Cancer cells are usually in a highly metabolically active state in order to maintain the nutrients they need to reproduce. Cancer cells have a highly oxidized state within them, and theoretically oxidative stress therapy should have better access for cancer, but in fact it is due to higher intracellular levels of GSH as a result of more cysteine intake by the cells, which makes the cancer cells more resistant to the highly oxidized environment, as well as more resistant to ferroptosis. When we use GSH inhibitors we can reverse the ferroptosis resistance of cancer cells and increase ferroptosis sensitivity to treat tumors. GSH metabolism can be involved in affecting lipid peroxidation levels within ferroptosis, mitochondrial morphology, alteration of ferroptosis marker genes, and mobilization of *TfR1*. However, the relationship between GSH metabolism and ferroptosis is still in doubt, and it is now known that saturated fatty acids (SFA) do not affect ferroptosis, monounsaturated fatty acids (MUFA) inhibit ferroptosis, and polyunsaturated fatty acids (PUFA) promote ferroptosis. The extent of GSH intervention on these three lipid peroxides, or their sensitivity and specificity, still needs to be further refined using targeted experiments to verify results. The next question is whether GSH has an effect on the ab initio synthesis of lipid peroxides. Iron metabolism is an important element in the maintenance of homeostasis and a key link in the occurrence of ferroptosis, and the effect of GSH metabolism on intracellular iron levels remains a mystery. Blue color means cells with GSH levels resistant to ferroptosis, while red color means cells with low GSH levels sensitive to ferroptosis. The triangular symbols imply mutations in the genes, while the arrows with question marks indicate that the relationship is not yet clear.

Numerous studies have consistently shown that the regulation of intracellular GSH levels can impact the initiation of ferroptosis in cancer cells and influence tumor development. It is noteworthy that while GSH deficiency can enhance ferroptosis in cancer cells and impede tumor progression, it is also associated with various other forms of programmed cell death, including apoptosis, necrosis, and autophagy. Additionally, GSH metabolism is closely related to a variety of tumors, especially concerning the development of tumors with oxidative stress damage as the main etiology [98] (Figure 6).

The following focuses on novel strategies for intervening in tumor therapy by mediating ferroptosis by major targets affecting GSH metabolism, mainly including SLC7A11, GCL, GSS, GGT, CD44, MRP1/2, and Chac1/2 (Table 1).

### 4.1. SLC7A11

As outlined in the discussion of GSH metabolic pathways, targeting different pathways of GSH metabolism can be utilized to modulate in vivo GSH levels and subsequently impact GSH-mediated resistance to iron-induced cell death. The functionality of the Xc-system may play a role in determining the clinical prognosis of tumors through its influence on intracellular cysteine levels, which serve as a precursor for GSH production [99]. Increased levels of SLC7A11 have been observed in multiple types of cancer and have been linked to resistance to chemotherapy and unfavorable outcomes in cancer patients. Suppression of SLC7A11 results in reduced uptake of extracellular cystine, leading to intracellular cysteine depletion and finally causing ferroptosis in cancer cells [100]. Consequently, the regulation of SLC7A11 expression is regarded as a potential therapeutic target for cancer treatment. Erastin, initially identified as an inducer of ferroptosis, was believed to primarily target mitochondrial voltage-dependent anion channels 2 and 3 (VDAC2 and VDAC3) [101]. Further research has shown that erastin acts as a strong inhibitor of the Xc-system, resulting in GSH depletion and subsequent initiation of ferroptosis [102,103]. The cell death induced by erastin can be prevented by supplementing the culture medium with GSH or N-acetylcysteine (NAC), a precursor of GSH biosynthesis [104]. Imidazole ketone erastin (IKE), a derivative of erastin, demonstrates inhibitory effects on the Xc-system and exhibits enhanced biological activity and tumor suppression [105]. Sorafenib, a multikinase inhibitor commonly utilized as a first-line treatment for advanced hepatocellular carcinoma, has been identified as a promoter of ferroptosis in hepatocellular carcinoma through Xc-system inhibition leading to glutathione depletion [106,107,108,109]. Sulfasalazine, an anti-inflammatory medication prescribed for inflammatory bowel disease and rheumatoid arthritis, has also been demonstrated to effectively inhibit the Xc-system [110]. Statins have been shown to sensitize cancer cells to both chemotherapy and radiation therapy, in addition to being effective in the treatment of dyslipidemia and reducing the occurrence of cardiovascular and cerebrovascular events [111,112]. The inhibition of HMG-CoA reductase by statins results in decreased GPx4 expression, leading to lipid ROS generation and the induction of ferroptosis in tumor cells [113,114]. 

Certain small molecule monomers have demonstrated the ability to regulate SLC7A11 activity, thereby impacting intracellular GSH levels and potentially offering therapeutic benefits for specific tumors. This suggests the possibility of these monomers serving as novel inducers of ferroptosis by modulating GSH content. Pseudolaric acid B (PAB), a natural diterpene acid derived from the roots and bark of Kaempferia, has been shown to induce ferroptosis in glioma cells by depleting cellular GSH through inhibition of SLC7A11. The inhibition of the Xc-system’s expression or function by PAB results in the retardation of tumor growth in vivo, as well as the suppression of cancer cell invasion and metastasis. This inhibitory effect on cancer cells is primarily due to the rapid depletion of GSH resulting from dysfunction of the SLC7A11 transporter, leading to lipid ROS accumulation and induction of ferroptosis [115]. Ursolic acid, a pentacyclic triterpenoid compound derived from traditional plants, when combined with sorafenib, has been shown to markedly enhance the accumulation of ROS in a hepatocellular model. This effect may be attributed to the downregulation of SLC7A11 expression, leading to a decrease in intracellular GSH levels and subsequent impairment of ROS scavenging capabilities. Consequently, lipid peroxidation is induced, finally inhibiting ferroptosis and yielding a notable therapeutic benefit in the treatment of hepatic cells [116]. Sodium butyrate demonstrates inhibitory effects on endometrial cancer cells and subcutaneous xenograft tumors through modulation of the GSH/GSSG ratio, intracellular ROS levels, and lipid peroxide content, thereby inducing ferroptosis in endometrial cancer cells [117]. Additionally, sodium butyrate exhibits potential in suppressing osteosarcoma growth and metastasis by promoting ferroptosis. Pretreatment with sodium butyrate exacerbates erastin-induced alterations in GSH depletion, lipid peroxidation, and mitochondrial morphology in CRC cells [118]. In a mechanistic manner, sodium butyrate was found to downregulate the transcription of SLC7A11 by modulating the expression of ATF3 [119]. Tirapazamine (TPZ) is a hypoxic prodrug known for its potent antitumor effects within the hypoxic microenvironment of tumors. TPZ demonstrated significant inhibitory effects on all three osteosarcoma cell lines. Furthermore, TPZ was observed to enhance fluorescent staining of ferrousb ions, while concurrently reducing the expression of SLC7A11 and GPx4, thereby promoting ferroptosis and inhibiting the proliferation and migration of osteosarcoma cells [120]. Pientzehuang (PZH) has been found to exhibit inhibitory effects on the diethylnitrosamine (DEN)-induced hepatocellular carcinoma (HCC) model in rats. The SLC7A11/GSH/GPx4 axis associated with the ferroptosis response is considered a potential target of PZH in mitigating the malignant transformation from liver fibrosis to HCC [121]. Levobupivacaine, a local anesthetic, also demonstrates potential anticancer properties by inducing ferroptosis in gastric cancer cells through the miR-489-3p/SLC7A11 axis, thereby impeding the proliferation of gastric cancer cells [122]. Curcumin, a polyphenol derived from turmeric, a traditional Chinese medicine, has been found to induce iron overload, GSH depletion, and lipid peroxidation in the curcumin-treated group with non-small cell lung cancer (NSCLC). Conversely, inhibition of Fer-1, a suppressor of ferroptosis, and iron response element binding protein 2 (IREB2) markedly reduced the anticancer effects and ferroptosis induced by curcumin in A549 and H1299 cells [123]. The natural compound β-elemene has been identified as a novel inducer of ferroptosis, and its combination with cetuximab treatment has shown sensitivity towards KRAS-mutant colorectal cancer (CRC) cells by promoting ferroptosis [124]. This finding is expected to offer potential therapeutic support for KRAS-mutant CRC cells. Agrimonolide, isolated from lungwort, exhibits diverse biomedical properties, including anticancer effects. In A2780 and SKOV-3 cells, agrimonolide demonstrated dose-dependent inhibition of proliferation, migration, and invasion, while also inducing apoptosis. Agrimonolide was found to induce ferroptosis in ovarian cancer cells, as demonstrated by increased levels of ROS, total iron, and Fe^2+^, as well as decreased expression of ferroptosis markers SLC7A11 and GPx4 [125]. Cisplatin (DDP) is a front-line therapy for advanced non-targeted NSCLC. Ginkgetin synergized have been shown to enhance the cytotoxic effects of DDP in NSCLC cells by promoting the accumulation of labile iron pools and lipid peroxidation. The study confirmed the involvement of ginkgetin synergized in mediating the ferroptosis process in NSCLC cells through a significant decrease in the expression of SLC7A11 and GPx4, as well as the GSH/GSSG ratio [126]. Mustardine (SI) was identified as a potent natural product with anti-NSCLC properties, inducing ferroptosis by increasing subferric iron content, ROS, and lipid peroxidation in cells. Additionally, SI treatment resulted in SLC7A11-dependent P53 downregulation [127]. Colorectal cancer stem cells (CCSC) are recognized for their significant impact on prognosis, chemotherapy resistance, and treatment outcomes in CRC. The administration of vitamin D resulted in a notable suppression of CCSC proliferation and a decrease in the quantity of tumor spheroids in an in vitro setting. Additional analysis revealed that vitamin D-treated CCSC displayed elevated levels of reactive oxygen species (ROS) and diminished levels of cysteine and GSH, along with thicker mitochondrial membranes. Further investigation indicated that SLC7A11 may be a specific target of vitamin D [128]. Tanshinone IIA, a bioactive compound derived from Salvia miltiorrhiza, was found to diminish the stemness of gastric cancer cells. Tanshinone IIA mechanistically induced an increase in lipid peroxidation levels and a decrease in ferroptosis markers in gastric cancer cells [129]. Ginsenoside Rh3 demonstrated efficacy in eliminating CRC cells by activating Gasdermin D (GSDMD)-dependent pyroptosis and inhibiting SLC7A11 through the Stat3/p53/NRF2 axis to induce ferroptosis [130]. Licorice chalcone A (Lico A), a significant constituent of traditional Chinese medicine Glycyrrhiza glabra, is a natural small molecule drug with diverse pharmacological properties. In vivo and in vitro studies have demonstrated that Lico A facilitates ferroptosis in hepatocellular carcinoma cells by suppressing the expression of SLC7A11, leading to the inhibition of the GSH-GPx4 pathway and the initiation of ROS activation [131]. Talaroconvolutin A (TalaA) has been identified as a novel inducer of ferroptosis, exhibiting dose- and time-dependent cytotoxicity against colorectal carcinoma cells. Notably, TalaA elevates reactive oxygen species levels to a critical threshold, beyond which ferroptosis is triggered. In contrast, the compound resulted in the downregulation of SLC7A11 channel protein expression while upregulating ALOX3, thereby facilitating ferroptosis [132]. Several investigations have demonstrated that saikosaponin A induces ferroptosis in HCC cells both in vitro and in vivo. Through RNA sequencing analysis, it was determined that saikosaponin A primarily impacts the glutathione metabolic pathway and suppresses the expression of the cystine transporter protein SLC7A11. Saikosaponin A was observed to elevate intracellular malondialdehyde (MDA) and iron levels, while concurrently reducing levels of reduced glutathione in HCC cells. Deferoxamine (DFO), Fer-1, and GSH were able to mitigate the cytotoxic effects of saikosaponin A, whereas Z-VAD-FMK was found to be ineffective in preventing saikosaponin A-induced cell death in HCC [133].

### 4.2. GCL and GSS

Aside from modulating ferroptosis through its impact on the Xc-system and subsequent regulation of raw material uptake for glutathione (GSH) synthesis, various other molecules have the potential to influence cellular sensitivity to ferroptosis by modulating GSH levels through alternative mechanisms. 

As previously stated, GCL serves as the rate-limiting enzyme in the biosynthesis of GSH and is essential for the synthesis and upkeep of cellular GSH. Upregulation of GCL leads to elevated levels of cellular GSH and enhances cellular resistance to oxidative stress. Conversely, downregulation of GCLC results in heightened accumulation of cellular lipid peroxides and increased sensitivity to ferroptosis [134,135]. In contrast, BSO, acting as a GCL inhibitor, has been demonstrated to trigger ferroptosis in tumor cells, specifically hepatocellular carcinoma, and has been observed to augment the effectiveness of nifurtimox in cancer cells, thereby serving as a significant regulator of GSH-mediated chemotherapy resistance [135]. However, BSO’s role as a modulator of ferroptosis in tumors is complex. Early clinical trials have shown that continuous infusion of BSO leads to decreased glutathione levels in tumors yet fails to demonstrate clinical efficacy. Cancer cells exhibiting sensitivity to GCLC inhibitors and characterized as sensitive demonstrated a significant response to BSO [136]. Increased levels of GCL/GSH have been implicated as the primary factor in resistance to cisplatin (DDP), and overexpression of GSH through transfection with an expression plasmid containing *GCLC* cDNA has been shown to enhance sensitivity to DDP by upregulating human copper transporter proteins [137]. Microcystin-LR (MCLR) is a potent hepatotoxic compound, and its oncogenic mechanism has been linked to a decrease in the expression and function of GCLC, resulting in sustained depletion of GSH levels and DNA damage induced by oxidative stress [138].

### 4.3. CD44/CD44v

GSH content is also regulated by CD44v, which affects the stability of the xCT conformation. CD44 is a cell surface glycoprotein that is overexpressed in a variety of cell types, including cancer stem cells, and CD44v is a variable shear form of CD44s. CD44 and its variants are highly expressed in a wide range of malignant tumors, and CD44v is strongly associated with tumor proliferation, invasion, metastasis, and drug resistance [139]. One study found that CD44v is heterogeneously expressed in mouse gastric tumors and is abundant in proliferating cells and slow-cycling stem cell-like cells [140]. Mechanistically, CD44v can promote an increase in GSH synthesis, a cystine uptake agent in tumor cells, by interacting with and stabilizing the SLC7A11 subunit of System Xc-. In turn, increased GSH content leads to a stronger antioxidant system in cancer cells to protect against oxidative stress damage caused by ROS. High levels of CD44v directly lead to high levels of GSH, and high levels of GSH as well as increased expression of antioxidant enzymes can promote cancer cell survival and resistance to anticancer drugs [141]. There are also a number of studies targeting CD44/CD44v in full swing, with one finding that nanoparticles actively targeting the CD44 receptor with GSH-responsive activity are potent therapeutic agents for breast cancer [142]. Therefore, targeting CD44v controls the cancer cell redox microenvironment by modulating xCT-mediated cystine transport to make tumors more sensitive to current cancer therapies. Poor penetration of drug therapy in pancreatic cancer patients is often the cause of chemotherapy failure. The sNP@G/IR consisting of a hyaluronic acid shell and a GSH core encapsulating both gemcitabine and photothermolysis can actively and precisely target tumors due to the HA-targeting of CD44, which is subsequently degraded by hyaluronidase in the extracellular matrix from which GSH is released. This sNP@G/IR with a dual cascade reaction both eliminates tumor-resident intracellular bacteria and enhances drug delivery [143]. Notably, CD44 has been identified as a typical apparent marker for CSC, while CD44v has been associated with poor prognosis in a variety of cancers due to its more specific expression in CSC than CD44; e.g., CD44v9 can be used as a predictive marker for recurrence in early primary gastric cancer [144]. However, targeting tumor-specific CD44v strategies still requires a great deal of in-depth research.

### 4.4. MRP1

MRP1 serves as a key protein in the generation of drug resistance in tumor cells, and numerous basic and clinical development trials of drugs targeting MRP1 are currently underway.

It has been found that (R)-verapamil hydrochloride and (S)-verapamil hydrochloride have differential effects on the regulation of MRP1, specifically the S isoform induces apoptosis in MRP1-transfected BHK-21 cells and results in a more drastic decrease in intracellular GSH content than the R isoform. Overall, (S)-verapamil hydrochloride induced apoptosis in potentially drug-resistant tumor cells, whereas (R)-verapamil hydrochloride rendered MRP1 overexpressing cells more sensitive to chemotherapeutic drugs [145]. Biricodar regulates Pgp, MRP-1, and BCRP (R482) and has the potential to act as a clinical broad-spectrum MDR regulator for the treatment of malignancies in which these proteins are expressed [146]. SS was identified as a novel selective MRP1 inhibitor in the screen, while it significantly slowed down MRP1 overexpressing tumor growth when combined with vincristine [147]. A study using positron emission tomography for ABC transporter protein substrate screening found that Ko143 potently inhibited MRP1 but was not specifically selective for MRP2, which may enhance the efficacy of anticancer drugs [148]. Reversan, an inhibitor of MRP1 discovered more than 10 years ago, inhibits MRP1 6–8 times more efficiently than conventional drugs [149]. Chaetominine demonstrated potent inhibition of highly expressed MRP1 at both the mRNA and protein levels, while no significant alterations were observed in the mRNA levels of the drug transporter protein MDR1. Additionally, the inhibitory effects of chaetominine on MRP1 were contingent upon the suppression of Akt phosphorylation and nuclear Nrf2. In summary, chaetominine effectively overcame drug resistance by disrupting the PI3K/Akt/Nrf2 signaling pathway, resulting in reduced MRP1-mediated drug efflux and triggering Bax/Bcl-2-dependent apoptosis in ADR-resistant K562/Adr leukemic cell lines [150]. In an experiment to study the effect of multidrug resistance protein (MRP) on the efficacy of gemtuzumab ozogamicin (GO) in the treatment of acute myeloid leukemia (AML), the results showed that MK571 increased the cytotoxicity of GO on MRP1-positive cell samples, demonstrating that MRP1 may be an important target for the emergence of drug resistance in GO [151].

However, there are still questions that need to be answered, the first of which is whether the use of inhibitors can increase drug accumulation in cancer in a clinical setting without unacceptable drug toxicity. Secondly controlling when and where the drug is effluxed is also a huge challenge [152].

### 4.5. GGT and Chac

GSH content is not only affected by the synthesis pathway and substrate raw materials, but also by GSH catabolism, which can be regulated by increasing GSH consumption, either by intervening in the exogenous metabolism of the key enzyme GGT or the endogenous metabolism of Chac1/2 enzymes.

Ammonium ferric citrate (AFC) is frequently utilized as a dietary supplement for iron fortification [153]. Several research studies have confirmed the ability of AFC to induce oxidative stress damage in NSCLC cell lines, resulting in a reduction in their autophagic capacity and triggering a ferroptotic response. Additionally, these studies have identified the GPx4-GSS/GSR-GGT axis as a crucial target for the ferroptotic response induced by AFC [154]. Other small molecule inhibitors of GGT, including Acivicin [155], Nahlsgen [156], and OU749 [157,158], have been shown to decrease GSH levels and exhibit potential ferroptosis-inducing properties. However, these glutamine analogs are typically associated with higher toxicity levels and are not considered suitable for clinical use. Therefore, further exploration into the development of non-competitive GGT inhibitors through foundational research is warranted. Dihydroartemisinin (DHA), an artemisinin derivative, has been studied as a potential antitumor agent in primary liver cancer (PLC). Exposure of hepatocellular carcinoma cells to DHA results in the manifestation of typical characteristics of ferroptosis, including elevated levels of lipid reactive oxygen species and malondialdehyde, iron overload, and reduced activity or expression of GSH, GPx4, SLC7A11, and SLC3A2. The promoter activity of the *CHAC1* gene, which encodes a protein involved in the degradation of GSH, was found to be increased following treatment with DHA [159]. In gastric cancer, lower levels of *CHAC1* mRNA were observed in tumor tissues compared to adjacent non-tumor tissues, and this decrease was associated with a poorer prognosis for patients. Additionally, OP-B was shown to enhance GSH metabolism and induce ferroptosis in gastric cancer cells by upregulating CHAC1 expression levels [160]. Multiple myeloma (MM) is a hematologic malignancy characterized by elevated levels of proteasome activity. While bortezomib, a proteasome inhibitor, has demonstrated efficacy in improving the survival rates of MM patients, the development of acquired resistance poses a significant obstacle in the management of this disease. Preconditioning of MM cells with DHA/EPA prior to treatment with bortezomib resulted in a significant reduction in cellular GSH levels and modulation of metabolites and key enzymes involved in GSH metabolism. RNA sequencing data indicated that the NRF2 activated the transcription factors ATF3/4, leading to the upregulation of the *CHAC1* gene, which plays a crucial role in GSH degradation [161]. 

In conclusion, all small molecules or drugs affecting GSH metabolism targets can accelerate intracellular GSH depletion, resulting in lipid ROS accumulation causing cellular ferroptosis, thus inhibiting cancer cell proliferation and acting as cancer therapeutics.

**Table 1:** Ferroptosis inducers with potential tumor therapeutic effects based on GSH metabolic strategies.

Small Molecule Compounds	GSH Metabolic Chain	Target	Experimental Model	Diseases	References
Erastin	synthesize	SLC7A11	cells, animals	HCC, GC, CRC	[86,87,88,89,90]
IKE	synthesize	SLC7A11	cells, animals	HCC	[91]
Sorafenib	synthesize	SLC7A11	cells, animals, clinical trial	HCC	[92,93,94,95]
Sulfasalazine	synthesize	SLC7A11	cells, animals, clinical trial	NSCLC	[99,100]
PAB	synthesize	SLC7A11	cells, animals	glioma	[101]
Ursolic acid	synthesize	SLC7A11	cells, animals	HCC	[102]
Butyrate sodium	synthesize	SLC7A11	cells, animals	endometrioma, osteosarcoma	[103,104,105]
TPZ	synthesize	SLC7A11	cells, animals	osteosarcoma	[106]
PZH	synthesize	SLC7A11	cells, animals	HCC	[107]
Levobupivacaine	synthesize	SLC7A11	cells, animals	GC	[108]
Curcumin	synthesize	SLC7A11	cells, animals	NSCLC	[109]
β-Elemene	synthesize	SLC7A11	cells, animals	CRC	[110]
Agrimonolide	synthesize	SLC7A11	cells, animals	ovarian cancer	[111]
Ginkgetin synergized	synthesize	SLC7A11, GCL	cells, animals	NSCLC	[112]
SI	synthesize	SLC7A11	cells, animals	NSCLC	[113]
Vitamin D	synthesize	SLC7A11	cells, animals	CRC	[114]
Tanshinone IIA	synthesize	SLC7A11	cells, animals	GC	[115]
Ginsenoside Rh3	synthesize	SLC7A11	cells, animals	CRC	[116]
Lico A	synthesize	SLC7A11	cells, animals	HCC	[117]
Talaroconvolutin A	synthesize	SLC7A11	cells, animals	CRC	[118]
Saikosaponin A	synthesize	SLC7A11	cells, animals	HCC	[119]
BSO	synthesize	GCL	cells, animals, clinical trial	HCC	[120,121,122]
MCLR	synthesize	GCL	cells, animals	HCC	[124]
AFC	decomposition	GGT	cells, animals	NSCLC	[126]
OP-B	decomposition	CHAC1	cells, animals	GC	[132]
DHA	decomposition	CHAC1	cells, animals	HCC	[131]
Acivicin	decomposition	GGT	NA	NA	[155]
Nahlsgen	decomposition	GGT	NA	NA	[156]
OU749	decomposition	GGT	NA	NA	[157,158]
(R)-Verapamil hydrochloride	uptake	MRP1	cells, animals, clinical trial	multidrug-resistant tumor	[145]
Biricodar	uptake	MRP1	cells, animals	multidrug-resistant tumor	[146]
LY-402913	uptake	MRP1	cells, animals	multidrug-resistant tumor	[147]
Ko 143	uptake	MRP1	cells, animals	multidrug-resistant tumor	[148]
Reversan	uptake	MRP1	cells, animals, clinical trial	multidrug-resistant tumor	[149]
Chaetominine	uptake	MRP1	cells, animals	multidrug-resistant tumor	[150]
MK-571	uptake	MRP1	cells, animals	multidrug-resistant tumor	[151]

NA: not available.

## 5. Conclusions

GSH, as the most important antioxidant active substance in organisms, regulates the reduction of lipid peroxides and plays an important role in ferroptosis as an essential cofactor of GPx4. The content of GSH is not only affected by the synthesis of its enzymatic system, but also by transport proteins, such as the membrane receptor SLC7A11 [162] and MRP1 [74], which are found on the cell surface. The difference of intracellular GSH content directly affects the change of cellular ferroptosis susceptibility. When the GSH level is high, intracellular ROS is reduced and the cell is in the antioxidant specialty, while when the GSH level is lowered, the intracellular ROS level rises and the cell is in the state of oxidative stress. The imbalance of oxidative and reductive mechanisms in the cancer cells, resulting in the fatal accumulation of lipid peroxidation, is a prerequisite for the occurrence of ferroptosis. Therefore, targeting GSH is the core link and regulatory switch to induce ferroptosis.

## 6. Prospects and Perspectives

Over the past few years, our research has led to a more comprehensive comprehension of GSH synthesis and metabolism, with a particular focus on its role as a pivotal regulator in ferroptosis. The development of pharmaceutical agents targeting GSH metabolism holds significant importance in addressing metabolic disorders associated with ferroptosis, where GSH plays a crucial role. 

However, there are still some key questions about GSH that remain unanswered. First, the complete metabolic regulatory network of GSH has still not been fully revealed; are there other synthetic pathways for GSH? What is the distribution of GSH in subcellular organelles such as Golgi and lysosomes, and what are the physiological and pathological significance? Are there other enzymes that catabolize GSH? What is the role of GSH for monounsaturated fatty acids? What is the molecular mechanism by which GSH regulates cellular ferroptosis in the physiologic state? How can we target cancer cell ferroptosis but not normal cell ferroptosis? How can we distinguish and understand physiological ferroptosis? What could be the benefits of targeting ferroptosis instead of apoptosis/necrosis in the context of cancer? These questions are the challenges and opportunities in translating GSH-ferroptosis research into clinical practice.

The precise regulation of GSH concentration stands as a crucial aspect in the advancement of precision medicine. It is anticipated that future therapeutic interventions for ferroptosis, centered on modulating GSH levels, will prove beneficial for individuals afflicted with ferroptosis-related conditions.

## Data Availability

Not applicable.
